# Associated factors for visibility and width of retrobulbar subarachnoid space on swept-source optical coherence tomography in high myopia

**DOI:** 10.1038/srep36723

**Published:** 2016-11-09

**Authors:** Hua Fan, Hongjie Ma, Rulong Gao, Danny Siu-Chun Ng, Carol Y. Cheung, Shuangnong Li, Dezheng Wu, Shibo Tang

**Affiliations:** 1 Aier School of Ophthalmology, Central South University, Changsha, China; 2 Aier Research Institute of Ophthalmology, Changsha, China; 3 Shenzhen Aier Eye Hospital, Shenzhen, China; 4 Guangzhou Aier Eye Hospital, Guangzhou, China; 5 Department of Ophthalmology & Visual Sciences, the Chinese University of Hong Kong, Hong Kong, China; 6 Zhongshan Ophthalmic Center, Sun Yat-sen University, Guangzhou, China

## Abstract

Subarachnoid space (SAS) around optic nerve can be visible with swept-source optical coherence tomography (SS-OCT). However, the relevant factors for its visibility and width have not been reported. In this prospective study, 193 eyes with high myopia were evaluated by SS-OCT. The relationship between age, gender, axial length, optic disc area, parapapillary atrophy (PPA) area, peripapillary choroidal thickness with the visibility and width of SAS were assessed. The results showed that SAS was observed in 125 (64.8%) and not observed in 68 (35.2%) eyes. Visibility of SAS is associated with long axial length, high myopia, thin choroid, large PPA and large optic disc areas. Among these associations, PPA area was the only independent factor (b = 0.177, p < 0.001). The width of SAS was associated with thin choroid, long axial length, large optic disc area and large PPA area. Multivariant analysis showed that optic disc area and PPA area were independent factors for the width of SAS (b = 30.8, p = 0.016 and 16.2, p < 0.001 respectively). These results suggested that SAS was extended into the peripapillary region possibly due to extension of posterior sclera in high myopia.

The optic nerve is enveloped by meninges and surrounded by retrobulbar subarachnoid space (SAS). Cerebrospinal fluid flows within the subarachnoid space between the arachnoid mater and the pia mater. In previous studies, SAS and the pressure of this fluid space were speculated to be related to many ocular diseases, including papilledema^1^, normal tension glaucoma^2^ and optic pits^3^. In a study of SAS in myopic eyes^4^,^5^, Jonas *et al*. reported that the thickness of scleral flange (sclera between the optic nerve border and the optic nerve dura matter) decreased significantly with axial length and the SAS was suspected to be dilated in highly myopic eyes. There were also several case reports in which intravitreous silicone oil migrated into the ventricles^6^,^7^,^8^,^9^. The mechanism of how silicone oil went into cerebral ventricle is still unclear, but the most likely access is through the SAS surrounding the optic nerve.

Traditional methods to access SAS include histology, magnetic resonance imaging and computer tomography. Nevertheless, histology has limited value because it can only examine enucleated eyes. The resolutions of magnetic resonance imaging and computer tomography are low. Optical Coherence Tomography (OCT) is a non-invasive technology providing *in vivo*, high resolution imaging for ocular structures. However, imaging the SAS using time domain and spectral domain OCT is sometimes difficult because the light signal is attenuated through the deep structures of the eyes, thus, obscuring the visibility of structures around the optic nerve. Swept-source OCT uses a frequency swept laser as a light source^10^ and has less roll-off of sensitivity with tissue depth than conventional Spectral Domain-OCT. Ohno-Matsui et cl reported that SAS could be seen by swept-source OCT in 93.2% of highly myopic eyes with an axial length more than 28 mm, but couldn’t be seen in any emmetropic eyes^4^. However, the relevant factors which affect the visibility of SAS nor its width have not been investigated.

The purpose of this study is to identify the ocular factors associated with the visibility and width of SAS from swept-source OCT in highly myopic eyes.

## Results

**Figure 1:**
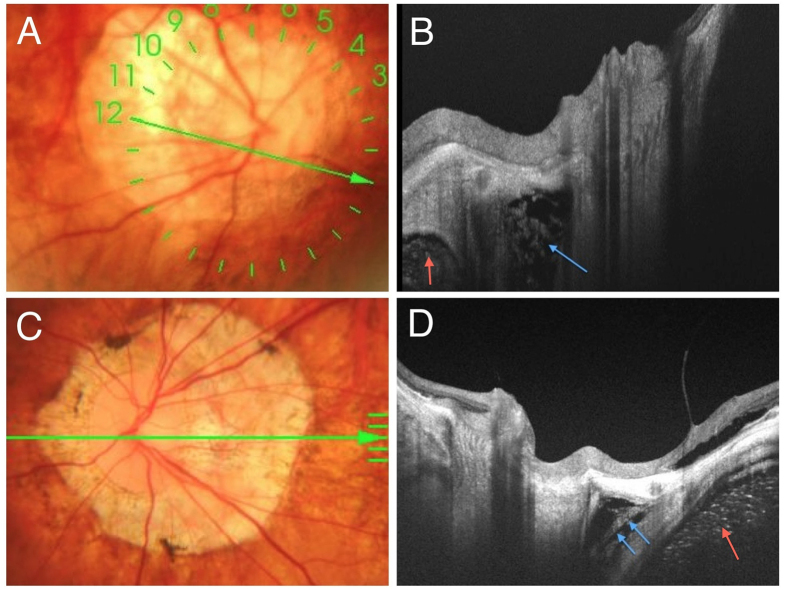
Fundus photographs and swept-source (SS) optical coherence tomography (OCT) images of both the subarachnoid space (SAS) (blue arrows) and the orbital fat (red arrows) next to the optic nerve in eyes with high myopia. (**A**) Color fundus photograph of the optic disc with an annular conus. Green arrow shows the locations of the OCT scans of (**B**). (**B**) Swept-source OCT slice scan along the green line in (**A**) shows the arachnoid trabeculae as multiple dots inside the hyporeflective area which is the SAS. (**C**) Color fundus photograph of the optic disc with a large myopic conus. Green arrow shows the location of the OCT scan of (**D**). (**D**) SS OCT B-scan image shows the arachnoid trabeculae as linear streaks. Orbital fat is observed as grayish tissue with many uniform dots in (**B,D**) (red arrow). The orbital fat is more hyperreflective and homogenous than the SAS.

One hundred ninety-three eyes of 105 consecutive patients with high myopia were included in this study. We found that only 64.8% of eyes (n = 125) had visible SAS from swept-source OCT images. The SAS was hyperrelective and appeared adjacent to the optic nerve just posterior to the globe. SAS was easier to be identified within the conus area. SAS was always observed temporal to the ON in eyes with large temporal conus. while SAS was observed on both sides of the optic nerve in eyes with annular atrophy. SAS was triangular with hyperreflective dots and lines inside, representing the trabeculae structures (Fig. 1). In some images, orbital fat was visible as a grayish tissue area with many relative hyperreflective dots, adjacent to the SAS and optic nerve through the dura mater. (Fig. 1) The hyperreflective dots had a more uniform structure, which helped to differentiate it from the trabeculae structures within SAS.

Comparison of the parameters between the eyes with and without visible SAS is shown in Table 1. There was no significant difference in age between the two groups. Although the eyes with visible SAS had worse best corrected visual acuity (BCVA) compare to those without, the difference was not statistically significant. The eyes with visible SAS had longer axial length, higher degree of myopia, thinner choroid and larger parapapillary atrophy (PPA) and optic disc area (all p < 0.05). There was more annual PPA in the eyes with SAS visible than those without. In the multivariate analysis, PPA area was the only independent factor significantly associated with the visibility of SAS (beta = 0.177, 95% CI: 0.035–0.318, p = 0.014), after adjusting for age, gender, axial length, choroid thickness, BCVA, spherical equivalent, optic disc area and inter-eye correlation.

**Figure 2:**
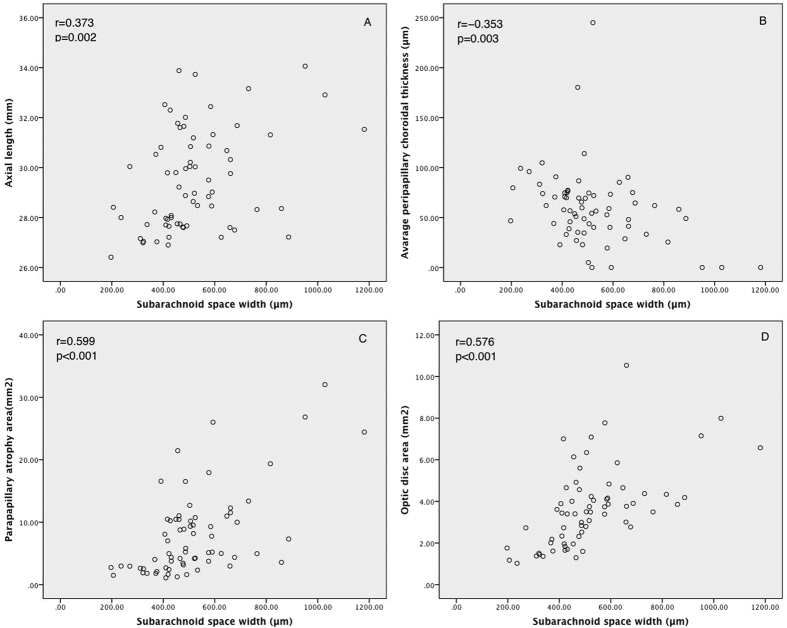
Scatterplot shows the correlation between axial length (**A**) peripapillary choroidal thickness (**B**) optic disc area (**C**) parapapillary atrophy area (**D**) and subarachnoid space width.

**Figure 3:**
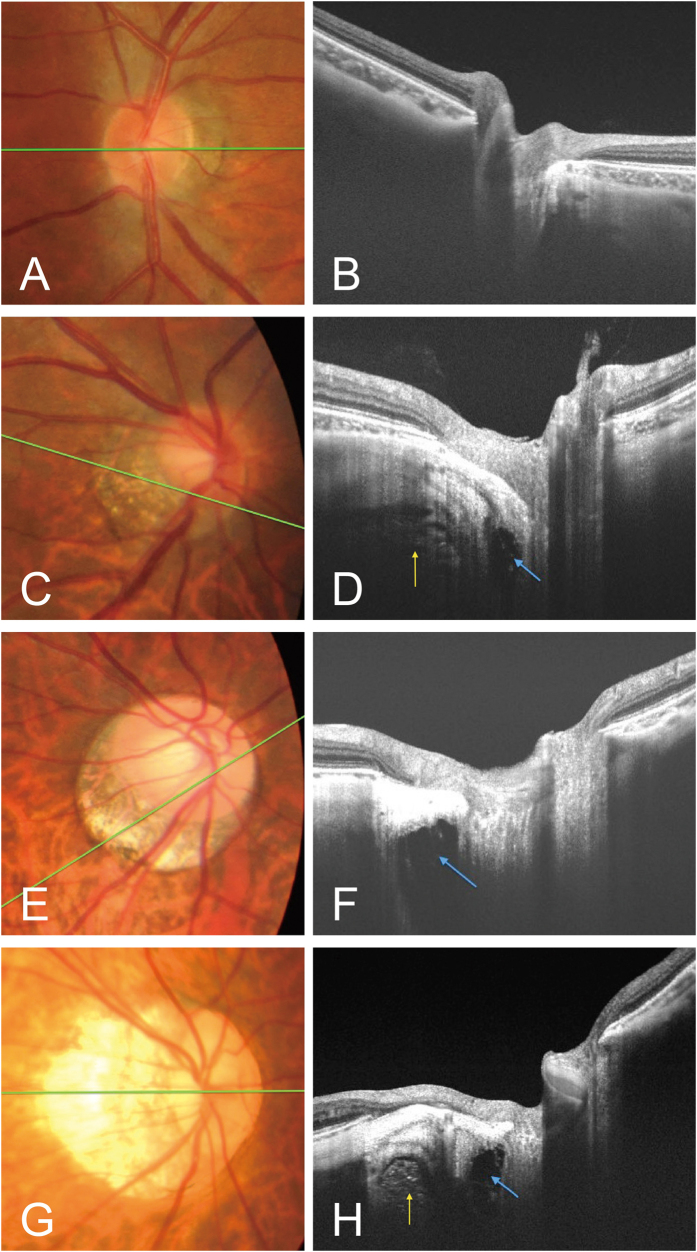
Swept-source (SS) optical coherence tomography (OCT) images of subarachnoid space (SAS) in eyes with various axial lengths. the axial length of (**A,B**) is 26.25, (**C,D**) is 26.72, (**E,F**) is 28.36, (**G,H**) is 30.21; Green line shows the OCT slice scan of the optic nerve, blue arrow shows the SAS, and yellow arrow shows the orbital fat. When the trabecular structure is not shown (**B**) we defined such images as SAS not visible.

The relationship of SAS width and various parameters are shown in Fig. 2. The width of SAS was not associated with age, gender or BCVA, mildly associated with average peripapillary choroidal thickness (r = −0.353, p = 0.003) and axial length (r = 0.373, p = 0.002). Moreover, it was moderately associated with optic disc area (r = 0.576, p < 0.001) and PPA area (r = 0.599, p < 0.001). In the multivariate analysis, both optic disc area (beta = 30.8, 95% CI: 5.72–55.9, p = 0.016) and PPA area (beta = 16.2, 95% CI: 7.82–24.5, p < 0.001) were the independent factors significantly associated with SAS width, after adjusting for age, gender, axial length, choroidal thickness, spherical equivalent and BCVA. OCT images of eyes with various PPA areas and axial lengths were shown in Fig. 3. Although eyes with longer axial lengths had wider SAS, in eyes with severe myopia but smaller PPA, the width of SAS was also narrower (Fig. 3G,H).

## Discussion

In this study, we found that visibility of SAS was independently associated with the area of PPA, and the width of SAS was independently associated with optic disc area and PPA area.

Compared with Ohno-Matsui *et al*.’s study, the proportion of visible SAS in our study was lower (64.8% vs. 93.2%)^4^. The lower proportion of SAS-visible eyes in our study may result from the following reasons: firstly, the definition of high myopia was different between Ohno-Matsui *et al*.’s study (spherical equivalent more than 8.00D or an axial length more than 26.5 mm) and our current study (spherical equivalent more than 6.00D and axial length more than 26 mm). It is noted that SAS was easier to be identified in eye with elongated axial length as shown in our current study. Secondly, we defined the hyporeflective area around the optic nerve without trabeculae structures inside as artifact area. Because of the difficulty in differentiating SAS from the hyporeflective area without definite trabeculae structures, we excluded the eyes with doubtful appearance of SAS. Thirdly, all of our study subjects were Chinese in our study while some subjects in Ohno-Matsui’s study were Caucasians. Caucasians have less pigment, and likely better light signal penetration into deep tissue.

In Ohno-Matsui *et al*.’s study^4^, there were no statistically significant differences of refractive error and axial length between the two groups. Nevertheless, we found that SAS was more likely to be visible in more myopic eyes, elongated axial length, thinner choroid, larger PPA area and optic disc area. This discrepancy may be explained by different sample sizes. There were only 9 cases in the control group in Ohno-Matsui *et al*.’s study while there were 68 controls in this study with higher statistical power. Furthermore, we have identified that the area of PPA, but not axial length or refractive error, was independently associated with the visibility of SAS. Tissues with less pigment allows more light signal penetration during OCT, which explains why deeper anatomic structures in eyes underneath the PPA can be more readily visible and most of the SAS-visible eyes had thin RPE and choroid.

A previous study reported that the width of SAS ranged from 263 to 1850 μm^4^. However, they did not report any factors associated with the width of SAS. Our study identified that the width of SAS was associated with optic disc area and PPA area in the multivariate analysis, suggesting that SAS width correlates with the expansion of posterior sclera, resulting in the expansion of nearby tissues, including optic nerve, PPA border and SAS. The observation that SAS is triangular in shape with its base towards the ocular wall also supports the expansion of posterior sclera at the junction of optic nerve and sclera. Jonas *et al*. studied the histology in 15 eyes with highly myopic peripapillary conus and found that the peripapillary region consisted of an elongation of the peripapillary scleral flange associated with extension of the retrobulbar cerebrospinal fluid (CSF) space into the retropapillary region^5^. Our *in vivo* imaging findings also support that the width of SAS was expanded with the extension of peripapillary structures in highly myopic eyes.

Xie *et al*. reported that the width of orbital SAS measured by magnetic resonance imaging correlated with intracranial pressure^11^. The orbital optic nerve SAS was narrower in patients with normal tension glaucoma compared to high pressure open angle glaucoma, suggesting that lower orbital CSF pressure in patients with normal tension glaucoma^12^,^13^. Glaucoma patients were also found to have larger PPA^12^,^13^. Further study is needed to investigate the visibility and measurement of SAS using high penetration OCT in patients with glaucoma. Since SS-OCT provides higher resolution images compared to magnetic resonance imaging, examination of more structural details will be available.

Our study had some limitations. First, we did not identify cases with glaucoma in our series since no visual field tests were performed. Second, it is noted that optic disc is tilted in most of myopic eyes and thus the measured area of the optic disc may be smaller from the en face images. Third, even with SS-OCT, SAS was not visible in some cases and the structure and measurements of SAS were not available. Further advances in OCT technology may provide better penetration for investigation of deep tissue in the future.

In conclusion, we used SS-OCT to observe and measure the SAS in proximity to the optic nerve in high myopia. The visibility of SAS was associated with larger PPA area. The width of SAS was associated with the areas of PPA and optic disc. Our findings suggested that SAS was extended into the peripapillary region possibly due to posterior sclera expansion in highly myopic eyes.

## Methods

This prospective study was approved by the Institutional Review Board of the Aier School of Ophthalmology, Central South University. The methods were carried out in accordance with the tenets of the Declaration of Helsinki. Patients with high myopia were recruited at the Retina Department, Guangzhou Aier Eye Hospital (Guangzhou, China) between April 8^th^, 2014 and February 1^st^, 2015. Written informed consent was obtained from all participants. The definition of high myopia was spherical equivalent >−6.00 diopters (D) and an axial length >26 mm. Patients with poor image quality (OCT image quality score < 40) because of dense cataract, vitreous hemorrhage, retinal detachment or poor fixation were excluded.

All participants underwent comprehensive ocular examination, including measurements of refractive error, BCVA, IOP, axial length measurements with coherence interferometry biometric measurement device (IOL Master; Carl Zeiss Meditec, Oberkochen, Germany), color photography of the fundus, slit-lamp biomicroscopy and detailed ophthalmoscopic evaluation. SS- OCT was performed in all included subjects with DRI-OCT (Topcon Corporation, Tokyo, Japan), which used a long wave length (1050 nm) light as its light source and has a scan speed of 100,000 A-scans per second. The axial resolution was 8μm and lateral resolution was 10 μm. The imaging depth was 2.5 mm. Four scanning protocols were used: 12 mm line scan cross the center of the optic disc and the fovea (1024 pixels); 12×9 mm^2^ three-dimensional (3D) scan covered the optic disc and macula using a resolution of 512(horizontal) ×256(vertical) pixels; 6×6 mm^2^ three-dimensional (3D) scan centered on the optic disc using a resolution of 512 (horizontal) ×128(vertical) pixels; and 9 mm radical line scan (12 lines) centered on the optic disc using a resolution of 1024 pixels in each line.

**Figure 4:**
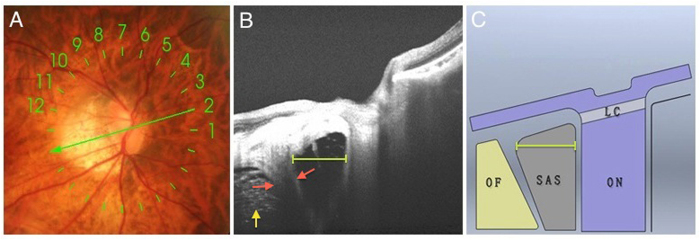
Measurement of the SAS width. (**A**) Color fundus photograph showing 9mm radical line scan (12 lines) centered on the optic disc. (**B**) Swept-source OCT slice scanned along the green arrow in (**A**) showing measurement of the SAS width in OCT image. SAS width was defined as the maximum distance between the dura mater (red arrow) and the optic nerve (yellow line). (**C**) The schematic drawing of (**B**). OF, orbital fat; ON, optic nerve; LC, lamina cribrosa. Yellow arrow indicates the orbital fat.

Visibility of SAS was evaluated according to the following standards: first, the SAS was a hyporeflective area surrounded by optic nerve sheath and pia mater and located between the optic nerve and orbital fat. Second, there were hyperreflective dots and lines inside the SAS indicating trabeculae structures. Third, the SAS was triangular in shape with the base towards the ocular wall. The width of SAS was defined as the maximum horizontal distance between the optic nerve and the dura mater and was measured on the images of 12 radical line scans centered on the optic disc (Fig. 4A). And the maximum width among the 12 radical images was used for analysis. The evaluations of SAS visibility and measurements of SAS width were performed by a single author (H.F.) who was masked to the patients’ clinical information such as axial length, BCVA, etc.

**Figure 5:**
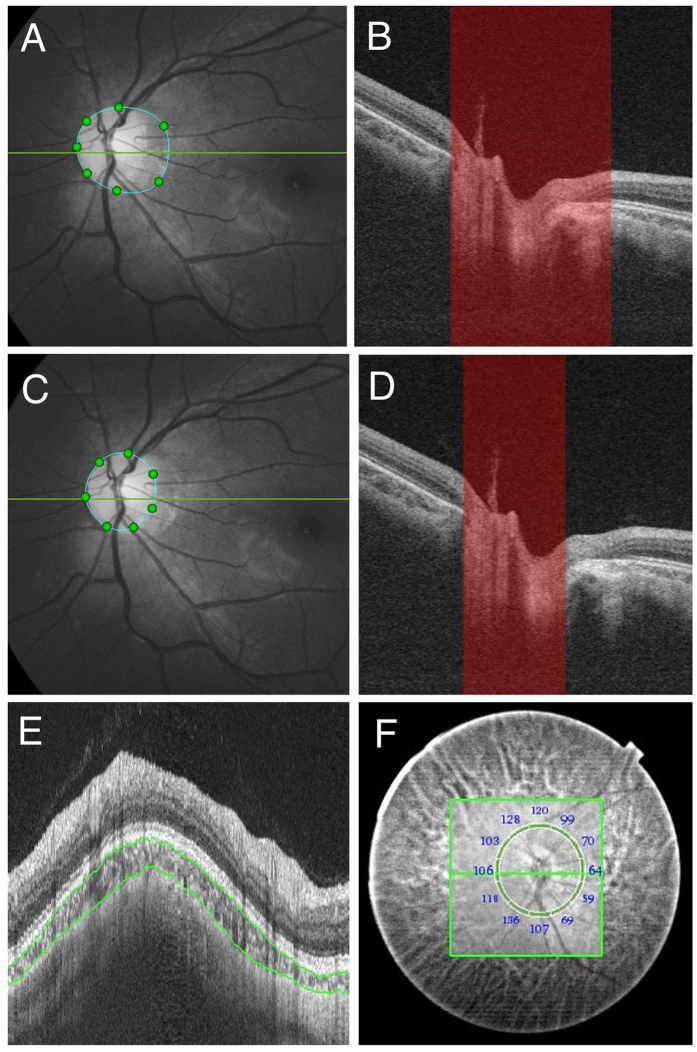
Measurement of parapapillary atrophy (**A–D**) and, peripapillary choroidal thickness (**E,F**). (**A,B**) show measurement of the parapapillary atrophy area combined with optic nerve area, (**C,D**) show measurement of the optic nerve area. Subtraction of optic nerve area from the combined area results in the parapapillary atrophy area.

The area of PPA, including the gamma zone combined with beta zone, was measured by the optic cup and disc measurement software of the SS- OCT as reported in literature^14^,^15^ (Fig. 5A–D). On the 12 × 9 mm^2^ three-dimensional (3D) scan, the border of optic disc and the border of PPA (gamma zone and beta zone, beginning of the Bruch’s membrane without retinal pigment epithelium) were marked. Areas of PPA and optic disc were then measured on the corresponding near-infrared reflectance images obtained by SS-OCT. The ocular magnification factor was corrected using the formula t = p × q × s, where “t” was the true scan length, “p” was the magnification related to the instrument, “q” was the magnification related to eye and “s” was the default scan length^16^. The value of “p” was 3.382 for the instrument calibrated axial length of 24.46 mm which was a constant for a telecentric imaging system. Ocular magnification factor “q” was calculated by the formula 0.01306 × (axial length- 1.82) where 1.82 was the distance between corneal apex and second principal point of the eye. Annular PPA was defined as a ring-shaped PPA surrounding the optic disc. Not-annular PPA was defined as temporal PPA or PPA that was superior or inferior to the optic nerve. The thickness of peripapillary choroid with diameter of 3.5 mm was measured using the planimetric caliper function of the built-in software of the SS-OCT (Fig. 5E,F).

Statistical analysis was performed using a commercially available statistical software package (SPSS for Windows, version 19.0, SPSS, Inc., Chicago, IL). The distribution of age, gender, AL, average peripapillary choroidal thickness, type of PPA, PPA area, optic disc area and corrected visual acuity were compared between the groups with and without PPA visible using the Mann-Whitney U test and chi-square test. *P* < 0.05 was considered significant. Generalized estimating equation adjusting for inter-eye correlation was performed to identify the independent factors associated with SAS visibility and width.

## Additional Information

**How to cite this article**: Fan, H. *et al*. Associated factors for visibility and width of retrobulbar subarachnoid space on swept-source optical coherence tomography in high myopia. *Sci. Rep*. **6**, 36723; doi: 10.1038/srep36723 (2016).

**Publisher’s note**: Springer Nature remains neutral with regard to jurisdictional claims in published maps and institutional affiliations.

## Figures and Tables

**Table 1:** Comparison of the eyes with and without subarachnoid space visible.

	Visible on OCT	Not Visible on OCT	Ρ
Number of eyes	125	68	
Age (year)	38.4 ± 13.9 (14–71)	34.2 ± 13 (19–71)	0.029*
Axial length (mm)	29.13 ± 2.06 (26.01–35)	28.15 ± 1.77 (26.04–34.84)	0.001*
Refractive error (Diopter)	13.46 ± 5.21 (6.00–27.75)	11.41 ± 4.63 (6.00–25.63)	0.006*
Peripapillary choroid thickness (μm)	63.40 ± 43.33 (0–245)	96.40 ± 59.75 (0–441.25)	<0.001*
PPA area (mm^2^)	8.07 ± 7.65 (0.65–38.17)	3.42 ± 4.02 (0.53–21.46)	<0.001*
Optic disc area (mm^2^)	3.42 ± 1.83 (1.03–10.63)	2.54 ± 1.15 (1.13–6.14)	0.001*
Visual acuity (LogMAR)	0.40 ± 0.60 (0–3)	0.27 ± 0.48 (0–2)	0.100*
PPA type (eye, %)			
Not Annular	64 (51.2%)	49 (72.1%)	0.005^#^
Annular	61 (48.8%)	19 (27.9%)	

PPA: parapapillary atrophy; *independent t test; ^#^chi-square test.
